# Severe Anaplasmosis With Multiorgan Involvement in a Rheumatoid Arthritis Patient

**DOI:** 10.7759/cureus.41536

**Published:** 2023-07-07

**Authors:** Yucel Aydin, Bhavya Vemuri, Syed M Ahmed, Mohamed Elgamal, Seyma Bilgin

**Affiliations:** 1 Department of Medicine, Saint Mary's Hospital, Waterbury, USA; 2 Department of Medicine, St. Mary's Hospital, Waterbury, USA

**Keywords:** acute respiratory distress syndrome (ards), doxycycline, tick-borne diseases, anaplasma phagocytophilum, anaplasmosis

## Abstract

Anaplasmosis, caused by the tick-borne bacterium *Anaplasma phagocytophilum*, is an emerging infectious disease with a broad spectrum of clinical manifestations. Here, we present a case report of a 66-year-old Caucasian woman residing in Connecticut who exhibited severe anaplasmosis with multi-organ involvement. The patient, with a medical history of rheumatoid arthritis and hypothyroidism, presented with confusion, lethargy, fever, myalgia, generalized weakness, and poor appetite in May 2023. Laboratory investigations revealed pancytopenia, hyponatremia, elevated liver enzymes with mild hyperbilirubinemia, and lactic acidosis. A buffy coat smear analysis demonstrated basophilic intracytoplasmic inclusion bodies in the neutrophils, supporting the diagnosis of severe anaplasmosis. Prompt administration of doxycycline, the recommended treatment for anaplasmosis, was initiated. However, the patient subsequently developed acute respiratory distress syndrome (ARDS) necessitating heated humidified high-flow nasal cannula (HFNC) therapy. *Anaplasma* polymerase chain reaction (PCR) confirmed the presence of the bacterium in the patient's blood. Following doxycycline treatment, the patient demonstrated improvement in peripheral blood findings, resolution of ARDS, and complete neurologic recovery. This case underscores the potential severity and diverse clinical manifestations of anaplasmosis, highlighting the importance of early recognition, prompt diagnosis, and timely initiation of appropriate treatment to prevent severe complications and improve patient outcomes.

## Introduction

Anaplasmosis is an emerging tick-borne zoonotic disease caused by the obligate intracellular bacterium *A. phagocytophilum*. It is primarily transmitted through the bites of infected black-legged ticks, including *Ixodes scapularis *and *Ixodes pacificus*. Humans can acquire the infection during outdoor activities in endemic areas, such as wooded and grassy regions. The prevalence of anaplasmosis varies from year to year and can be found in various states in the United States. However, anaplasmosis is more common in certain regions including the upper Midwest, Northeast, and mid-Atlantic states. Connecticut is one of the states in the northeast region of the United States where anaplasmosis is more commonly reported. Since 2008, an average of 69 cases (range 22 to 120) have been reported in Connecticut annually. The statewide incidence rate is 3.1 per 100000 persons and it is more common between late spring and early fall with a peak in June.

Anaplasmosis primarily targets neutrophils, resulting in various clinical manifestations, including fever, myalgia, generalized weakness, and flu-like symptoms. These symptoms typically manifest within one to two weeks after a tick bite [1]. While some individuals, particularly those with intact immune systems, may remain asymptomatic or experience mild and self-limiting symptoms, immune-deficient patients are at a higher risk of developing severe forms of the disease with multi-systemic complications. Prompt and accurate diagnosis, followed by appropriate treatment, is crucial to prevent adverse outcomes, limit bacterial dissemination, and reduce the risk of complications [2].

Babesiosis, another tick-borne disease caused by protozoan parasites of the *Babesia microti*, can be observed as a co-infection with anaplasmosis at 5-20% depending on the prevalence of each infection. The transmission of both pathogens occurs through the bite of infected ticks, and therefore, simultaneous infections can occur in individuals exposed to ticks carrying both pathogens [3]. In this case report, we comprehensively describe a severe anaplasmosis case with multi-systemic involvement, encompassing neurological, respiratory, and hepatobiliary manifestations. The aim of this report is to highlight the diagnostic challenges faced in identifying and managing anaplasmosis with diverse clinical presentations, as well as to discuss the strategies employed for effective management. By elucidating the complexities associated with severe anaplasmosis, we aim to enhance the understanding of this emerging tick-borne disease and emphasize the importance of early recognition, accurate diagnosis, and timely initiation of appropriate treatment to optimize patient outcomes.

## Case presentation

A 66-year-old Caucasian woman residing in Connecticut presented to the emergency department with a two-day history of confusion and lethargy in May 2023. The patient had a significant medical history of rheumatoid arthritis for 20 years, managed with a weekly dose of methotrexate (20 mg), and daily administration of upadacitinib (15 mg). Additionally, she had hypothyroidism and received levothyroxine supplementation. On arrival, the patient was notably disoriented, history was taken from the patient's husband. According to her husband, the patient had experienced subjective fever, chills, generalized weakness, and poor appetite over the past two days. The patient has a history of gardening and contact with grasses and was found to have a couple of ticks in the left armpit 3 days earlier before the presentation. A review of the patient's symptoms did not reveal any additional remarkable findings. Concerned about worsening her mental status, the husband promptly contacted emergency services.

Upon examination, the patient's vital signs in the emergency department were as follows: temperature of 101.9°F, heart rate of 130 beats per minute, respiratory rate of 29 breaths per minute, and oxygen saturation of 85% on ambient air, which improved to 94% with the administration of 3 liters per minute of nasal cannula oxygen supplementation. The patient appeared obtunded and unresponsive to simple commands during the physical examination. Chest and heart examinations yielded unremarkable results, aside from tachycardia and tachypnea. Skin examination did not reveal any noticeable rashes. 

Laboratory analysis, as shown in Table 1, exhibited significant findings, including pancytopenia, hyponatremia, predominantly elevated aspartate transaminase (AST) liver enzyme levels with mild hyperbilirubinemia, and elevated lactic acid levels. SARS-CoV-2 RNA PCR was not detected for COVID-19 infection. 

**Table 1:** Trend of laboratory data after treatment. WBC: white blood cells; AST: aspartate aminotransferase; ALT: alanine aminotransferase

Laboratory Reference	On admission	Day 4	Day 7
WBC (4.0-10.5 k/µL)	3.2	1.9	11.2
Hemoglobin (13.5-18.0 g/dL)	11.7	8.5	8.5
Platelets (150-450 k/µL)	21	14	100
Creatinine (0.7-1.3 mg/dL)	0.8	0.8	0.5
Sodium (135-145 mEq/L)	132	142	143
Potassium (3.6-5.2 mmol/L)	3.1	3.8	3.1
AST (13-39 U/L)	520	607	378
ALT (7-52 U/L)	179	188	178
Total bilirubin (<1 mg/dL)	1.6	2.5	1.2
Albumin (3.4-5.4 g/dL)	4	2.7	2.8
Prothrombin time (10-13 secs)	18.8	11.7	12.5
Lactic acid (0.5-2.0 mmol/L)	2.4	1.6	Not done

**Figure 1:**
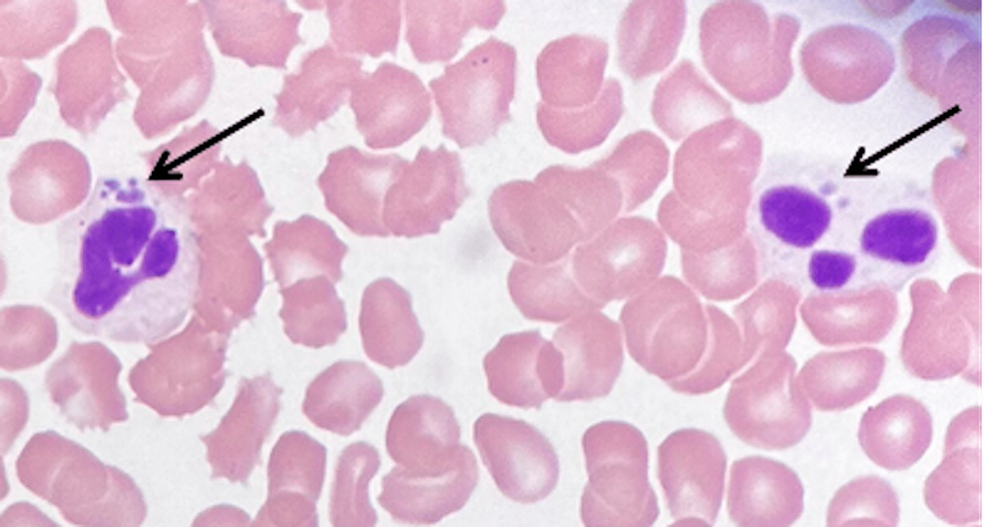
Buffy coat smear of the patient. Arrows indicate intracytoplasmic basophilic inclusion bodies in the neutrophils that are consistent with anaplasmosis. Images were taken under magnification x40.

A buffy coat smear analysis revealed the presence of multiple basophilic intracytoplasmic inclusion bodies, consistent with the clinical diagnosis of anaplasmosis (Figure 1).

**Figure 2:**
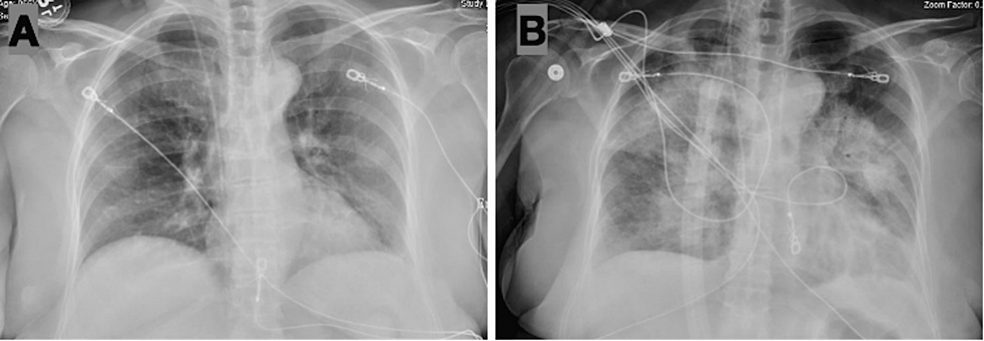
Comparison of the anterior-posterior chest X-rays on the day of admission (A) and on day 3 (B). A bilateral patchy infiltration is seen on day 3.

The patient was admitted to the medical intensive care unit and treatment with doxycycline 100 milligrams every 12 hours was initiated promptly. Magnetic resonance imaging (MRI) of the brain revealed no acute pathology. Due to having a clinical explanation of altered mental status with anaplasmosis, lumbar puncture was deferred. Her hospital course was complicated by the development of acute hypoxic respiratory failure secondary to acute respiratory distress syndrome (ARDS), necessitating heated humidified high-flow nasal cannula (HFNC) therapy with a flow rate of 40 liters per minute and 60% oxygen supplementation at day 3 of the treatment, as depicted in Figure 2. Urine legionella and streptococcal antigens remained negative, sputum culture grew normal pharyngeal flora. Echocardiography showed normal valvular function with an ejection fraction of 67%. 

Further diagnostic evaluation through *Anaplasma *PCR confirmed the presence of *A. phagocytophilum* infection, thus supporting the clinical diagnosis. Co-infection with *Babesia *or Lyme disease was excluded as the *B. microti *PCR and Lyme disease (*Borrelia burgdorferi*) antibodies Ig G and Ig M yielded negative results. Subsequent laboratory assessments demonstrated late-stage improvement (Table 1) following the administration of doxycycline, as evidenced by improvements in peripheral blood findings, neurologic findings, and resolution of ARDS at day 10. The patient was discharged on day 12.

## Discussion

Anaplasmosis can manifest with a wide range of symptoms, making it a challenging disease to identify. Common symptoms include fever, headache, muscle aches, fatigue, and chills, which are often mistaken for other common illnesses such as influenza. The absence of a clear tick bite history further complicates the diagnosis, as patients may not connect their symptoms to a tick exposure. Severe anaplasmosis is a highly concerning medical condition that involves the simultaneous dysfunction of multiple organ systems [4]. The disease manifests with neurological, respiratory, and hepatobiliary symptoms, presenting a diagnostic challenge. However, in the case presented, the presence of morulae on the buffy coat smear is suggestive of the diagnosis which will be later confirmed by the PCR. 

The timely initiation of appropriate treatment, specifically the administration of doxycycline, proved to be crucial in preventing further complications and promoting the patient's recovery. Severe complications may include respiratory failure, kidney failure, bleeding disorders, and even death in rare cases [5]. Therefore, prompt diagnosis and timely initiation of appropriate treatment are essential to prevent these complications and improve patient outcomes. Although the patient initially showed a delayed response to doxycycline for up to 5 days, subsequent improvements in peripheral blood findings and the resolution of severe anaplasmosis were observed. However, as a result of the disease's progression, the patient developed ARDS, necessitating the use of HFNC therapy to maintain adequate oxygenation. However, ARDS is a very unusual presentation of anaplasmosis that is rarely reported in the literature. The patient's eventual recovery from ARDS and subsequent discharge on day 12 with close follow-up underscore the importance of early recognition and prompt diagnosis in anaplasmosis cases. It should be noted that the patient was on methotrexate and upadacitinib, both of which are immunosuppressants - this likely contributed to the worsening of her symptoms and delayed response along with diverse clinical manifestations that affected multiple organ systems. 

Healthcare providers must maintain a high index of suspicion for anaplasmosis, particularly in areas where ticks are prevalent, such as wooded or grassy regions. *A. phagocytophilum* is primarily transmitted to humans through the bite of infected black-legged ticks (*I. scapularis* in the northeast and midwest and *I. pacificus* on the Pacific coast ) known as deer ticks. Improving awareness among healthcare professionals regarding the various presentations and complications of anaplasmosis can lead to earlier diagnosis and better outcomes for affected patients. In order to enhance patient care, further research is warranted to better comprehend the pathogenesis and optimize the management of severe anaplasmosis with multi-system involvement. Such investigations would contribute to developing more effective treatment strategies and preventive measures.

Preventing anaplasmosis primarily revolves around taking proactive measures to avoid tick bites. This includes wearing appropriate protective clothing such as long sleeves, long pants, closed-toe shoes, and hats when entering tick-endemic areas. Tucking pants into socks or boots can further minimize the risk of ticks attaching to the body. Additionally, using a United States Environmental Protection Agency (EPA)-registered insect repellents containing DEET (N,N-diethyl-meta-toluamide), picaridin, or permethrin on exposed skin and clothing can effectively repel ticks. It is essential to follow the instructions on the product label for proper application. After spending time in tick-infested areas, performing thorough tick checks on the body and removing any attached ticks promptly can help prevent transmission. Educating individuals living in or visiting tick-endemic regions about these preventive measures is crucial for raising awareness and promoting proactive behavior to reduce the incidence of anaplasmosis. Post-tick bite antibiotic prophylaxis is not recommended to prevent anaplasmosis. Individuals who have been bitten by a tick should watch for signs and symptoms. They should see their healthcare provider if fever, rash, or other symptoms develop within two weeks of the tick bite. [6]. 

## Conclusions

Severe anaplasmosis is a complex disease characterized by its multi-systemic involvement and the potential for severe complications. Early recognition, prompt diagnosis, and timely initiation of appropriate treatment, such as doxycycline, are essential in preventing further complications and improving patient outcomes. Increased awareness among healthcare providers, along with further research, will contribute to improved management and better outcomes for individuals affected by anaplasmosis. It highlights the need for healthcare providers to maintain a high index of suspicion, recognize the disease early on, and promptly initiate appropriate treatment. By doing so, severe complications can be prevented, and patient outcomes can be significantly improved. Additionally, raising awareness about preventive measures can help reduce the incidence of anaplasmosis in tick-endemic areas.

## References

[1] Ismail N, Bloch KC, McBride JW (2010). Human ehrlichiosis and anaplasmosis. Clin Lab Med.

[2] Dropulic LK, Lederman HM (2016). Overview of infections in the immunocompromised host. Microbiol Spectr.

[3] Rocha SC, Velásquez CV, Aquib A, Al-Nazal A, Parveen N (2022). Transmission cycle of tick-borne infections and co-infections, animal models and diseases. Pathogens.

[4] Dumic I, Jevtic D, Veselinovic M (2022). Human granulocytic anaplasmosis-a systematic review of published cases. Microorganisms.

[5] Thomas RJ, Dumler JS, Carlyon JA (2009). Current management of human granulocytic anaplasmosis, human monocytic ehrlichiosis and Ehrlichia ewingii ehrlichiosis. Expert Rev Anti Infect Ther.

[6] Eisen L (2022). Personal protection measures to prevent tick bites in the United States: knowledge gaps, challenges, and opportunities. Ticks Tick Borne Dis.

